# Optimizing administrative datasets to examine acute kidney injury in the era of big data: workgroup statement from the 15^th^ ADQI Consensus Conference

**DOI:** 10.1186/s40697-016-0098-5

**Published:** 2016-02-26

**Authors:** Edward D. Siew, Rajit K. Basu, Hannah Wunsch, Andrew D. Shaw, Stuart L Goldstein, Claudio Ronco, John A. Kellum, Sean M. Bagshaw, on behalf of the 15th ADQI Consensus Group

**Affiliations:** Tennessee Valley Health System (TVHS), Nashville Veterans Affairs Hospital, Nashville, TN USA; Vanderbilt University Medical Center, Department of Medicine, Division of Nephrology and Hypertension, Vanderbilt Center for Kidney Diseases (VCKD), 1161 21st Avenue South, MCN S3223, Nashville, TN 37232 USA; Cincinnati Children’s Hospital, Department of Pediatrics, Division of Critical Care Medicine and the Center for Acute Care Nephrology, The University of Cincinnati, Cincinnati, OH USA; Department of Critical Care Medicine, Sunnybrook Health Sciences Center Center and Sunnybrook Research Institute; Department of Anesthesia and Interdepartmental Division of Critical Care, University of Toronto, Toronto, ON Canada; Vanderbilt University Medical Center Department of Anesthesiology, Nashville, TN USA; Center for Acute Care Nephrology, Cincinnati Children’s Hospital Medical Center, Cincinnati, OH USA; Department of Nephrology, Dialysis and Transplantation, International Renal Research Institute (IRRIV), San Bortolo Hospital, Vicenza, Italy; Center for Critical Care Nephrology, Department of Critical Care Medicine, University of Pittsburgh, Pittsburgh, PA USA; Division of Critical Care Medicine, Faculty of Medicine and Dentistry, University of Alberta, Alberta, Canada

## Abstract

**Purpose of review:**

The purpose of this review is to report how administrative data have been used to study AKI, identify current limitations, and suggest how these data sources might be enhanced to address knowledge gaps in the field.

**Objectives:**

1) To review the existing evidence-base on how AKI is coded across administrative datasets, 2) To identify limitations, gaps in knowledge, and major barriers to scientific progress in AKI related to coding in administrative data, 3) To discuss how administrative data for AKI might be enhanced to enable *“*communication” and “translation” within and across administrative jurisdictions, and 4) To suggest how administrative databases might be configured to inform ‘registry-based’ pragmatic studies.

**Source of information:**

Literature review of English language articles through PubMed search for relevant AKI literature focusing on the validation of AKI in administrative data or used administrative data to describe the epidemiology of AKI.

**Setting:**

Acute Dialysis Quality Initiative (ADQI) Consensus Conference September 6-7^th^, 2015, Banff, Canada

**Patients:**

Hospitalized patients with AKI

**Key messages:**

The coding structure for AKI in many administrative datasets limits understanding of true disease burden (especially less severe AKI), its temporal trends, and clinical phenotyping. Important opportunities exist to improve the quality and coding of AKI data to better address critical knowledge gaps in AKI and improve care.

**Methods:**

A modified Delphi consensus building process consisting of review of the literature and summary statements were developed through a series of alternating breakout and plenary sessions.

**Results:**

Administrative codes for AKI are limited by poor sensitivity, lack of standardization to classify severity, and poor contextual phenotyping. These limitations are further hampered by reduced awareness of AKI among providers and the subjective nature of reporting. While an idealized definition of AKI may be difficult to implement, improving standardization of reporting by using laboratory-based definitions and providing complementary information on the context in which AKI occurs are possible. Administrative databases may also help enhance the conduct of and inform clinical or registry-based pragmatic studies.

**Limitations:**

Data sources largely restricted to North American and Europe

**Implications:**

Administrative data are rapidly growing and evolving, and represent an unprecedented opportunity to address knowledge gaps in AKI. Progress will require continued efforts to improve awareness of the impact of AKI on public health, engage key stakeholders, and develop tangible strategies to reconfigure infrastructure to improve the reporting and phenotyping of AKI.

**Why is this review important?:**

Rapid growth in the size and availability of administrative data has enhanced the clinical study of acute kidney injury (AKI). However, significant limitations exist in coding that hinder our ability to better understand its epidemiology and address knowledge gaps. The following consensus-based review discusses how administrative data have been used to study AKI, identify current limitations, and suggest how these data sources might be enhanced to improve the future study of this disease.

**What are the key messages?:**

The current coding structure of administrative data is hindered by a lack of sensitivity, standardization to properly classify severity, and limited clinical phenotyping. These limitations combined with reduced awareness of AKI and the subjective nature of reporting limit understanding of disease burden across settings and time periods. As administrative data become more sophisticated and complex, important opportunities to employ more objective criteria to diagnose and stage AKI as well as improve contextual phenotyping exist that can help address knowledge gaps and improve care.

## ABRÉGÉ

**Article synthèse:**

Cette revue vise à rendre compte de la manière dont les données administratives ont été utilisées jusqu’à maintenant pour l’étude de l’insuffisance rénale aiguë (IRA). On a également voulu définir les limites actuelles et suggérer une manière dont les sources de données pourraient être améliorées pour pallier les lacunes des connaissances dans ce domaine.

**Objectifs de la revue:**

Cette revue visait plusieurs objectifs:

1) Répertorier les données probantes sur la manière dont l’IRA est codée dans les ensembles de données administratives.

2) Identifier les limites, les lacunes au niveau des connaissances et les principaux obstacles aux avancées de la science relevant de la codification de l’IRA dans les données administratives.

3) Discuter de la façon dont les données administratives relatives à l’IRA pourraient être bonifiées afin de favoriser leur transmission au sein des différents secteurs de compétences.

4) Suggérer une nouvelle façon de configurer les bases de données administratives afin qu’elles puissent servir de source d’information pour la tenue d’études pragmatiques fondées sur la consultation de registres.

**Sources:**

L’information a été colligée à la suite d’un passage en revue des articles pertinents publiés en anglais sur PubMed. Les articles portant sur la validation de l’IRA au sein des données administratives ou sur l’usage de celles-ci pour rendre compte de l’épidémiologie de l’IRA ont été retenus.

**Cadre de la revue:**

La revue a eu lieu dans le cadre de la 15e réunion annuelle de concertation de l’*Acute Dialysis Quality Initiative* (ADQI) qui s’est tenue les 6 et 7 septembre 2015 à Banff, au Canada.

**Population observée:**

Les cas retenus pour la revue sont ceux de patients hospitalisés atteints d’IRA.

**Points saillants:**

La façon dont l’IRA est codée au sein des ensembles de données limite la compréhension réelle du fardeau que représente cette maladie, particulièrement pour les patients atteints moins sévèrement. Il semble également que l’on ne puisse identifier correctement les tendances temporelles ni le phénotype clinique de l’IRA parmi ces données. On a aussi constaté qu’il existe d’importantes possibilités d’amélioration de la codification de l’IRA dans les ensembles de données pour arriver à mieux cerner les lacunes dans la connaissance de la maladie et bonifier les soins prodigués aux patients.

**Méthodologie:**

Une version adaptée de la méthode Delphi pour l’atteinte d’un consensus a été utilisée pour produire le compte-rendu. La revue de la littérature et des récapitulatifs ont été élaborés à la suite d’une série de discussions alternant entre des ateliers en petits groupes et des séances plénières.

**Résultats:**

Les codes administratifs concernant l’IRA sont limités en raison du faible taux de rappel des documents pertinents lors des recherches, du manque de normalisation dans le classement de la sévérité de la maladie et de la faible identification de son phénotype. Ces limites sont de plus freinées par un manque de sensibilisation des fournisseurs de soins de santé face à l’IRA et par le caractère subjectif d’un signalement volontaire des cas observés. Bien qu’une définition idéalisée de l’IRA soit difficile à mettre en œuvre, améliorer la normalisation des signalements en se basant sur un consensus scientifique et fournir de l’information quant au contexte où se manifeste l’IRA sont possibles. Les bases de données administratives peuvent améliorer les pratiques entourant la conduite d’études cliniques ou d’études pragmatiques basées sur la consultation de registres, et servir de sources d’informations pour celles-ci.

**Limites de la revue:**

La provenance des sources consultées pour cette revue se limite en grande partie à l’Amérique du Nord et à l’Europe.

**Conclusions:**

La croissance et l’évolution rapide des bases de données administratives représentent une occasion unique de s’attaquer aux lacunes dans les connaissances sur l’IRA. L’avancement de ces connaissances demandera le déploiement d’efforts constants pour favoriser la prise de conscience des répercussions de cette maladie sur la santé publique. Cela demandera aussi l’engagement des principaux intervenants ainsi que la mise en place de stratégies concrètes visant la reconfiguration des infrastructures existantes et l’amélioration de la collecte de données et de l’identification du phénotype de l’IRA.

## Background

The clinical study of acute kidney injury (AKI) has been facilitated in recent years by the increasing availability of administrative data. The housing of vast amounts of information in accessible data warehouses and the relatively low cost of procurement suggest potential usefulness. However, as usually not collected for conducting clinical research, concerns over the quality of the data and its ability to fill knowledge gaps and evaluate quality of care have been raised [1, 2]. On September 6th, 2015, the Acute Dialysis Quality Initiative (ADQI) convened a panel of experts in nephrology, critical care, pharmacology, pediatrics, epidemiology, health services research and data analytics from five countries from North American and Europe to examine how rapidly evolving clinical data infrastructures in the era of ‘big data’ can be leveraged to enhance scientific progress and improve outcomes in patients with AKI. Here, we review how administrative data have been used to study AKI, identify current limitations, and suggest how these data sources might be enhanced to address knowledge gaps in the field. We present four key questions regarding the use of administrative data for research and quality improvement; and then a corresponding series of consensus statements developed through reviewing the literature and iterative discussion.

## Methods

The ADQI methodology has been previously detailed [3]. In brief, the consensus-building process was informed by pre-conference, conference, and post-conference review of English language articles through PubMed search for relevant AKI literature. We selected articles if they focused on the validation of AKI in administrative data or used administrative data to describe the epidemiology of AKI.

We conducted a 2-day conference in September 2015 in Banff, Canada, where summary statements were developed through a series of alternating breakout and plenary sessions. Panelists were assigned to 3–5 person workgroups. In each breakout session, the workgroups refined the key questions, identified the supporting evidence, and generated consensus statements. Workgroup members presented their findings during the plenary sessions and then revised their drafts as needed until a final version was agreed upon. A writing committee assembled the individual reports from the workgroups and each report was edited to conform to a uniform style and for length. The final reports were provided to each participant for comment.

## Review

*Question #1: What is the existing evidence-base for how AKI is coded across various administrative datasets?*

*Consensus Statement #1A: Administrative data are defined as information collected and stored for patient or disease registration, to inform transactions, or to promote other record keeping. The coding of AKI using these data sources has utilized billing/claims, limited laboratory-based definitions in disease-specific registries, and population-based health registries.*

*Consensus Statement #1B: Commonly used administrative codes for AKI generally demonstrate poor sensitivity and high specificity.*

For this manuscript, we will define health care-related administrative data as information collected and stored for patient disease registration, to inform transactions, or perform other record keeping (http://www.adls.ac.uk/adls-resources/guidance/introduction/).

Major producers of administrative data include, but are not limited to, governmental agencies (e.g., Federal, State/Provincial, and Veterans Affairs), insurers, and healthcare systems. Most literature using administrative data to study AKI have applied the 9^th^ and 10^th^ iterations of the International Classification of Disease, Clinical Modification (ICD-9-CM, ICD-10-CM) and procedure codes and the American Medical Association’s Current Procedural Terminology (CPT) codes to capture the diagnosis of AKI and renal replacement therapy (RRT) [2, 4]. Within these classification systems, AKI is most commonly coded as Acute Kidney Failure with various histologic descriptors (Table 1). The latter are generally based on clinical impression as confirmatory histologic diagnosis requires kidney biopsy, something rarely pursued in clinical practice. Further these histologic diagnoses have traditionally been non-exhaustive and include distinctions of less clear relevance (e.g. tubular versus medullary necrosis). While subsequent iterations (ICD-10) have broadened to capture additional lesions of interest (e.g. acute tubulo-interstitial nephritis), they remain independent codes that do not readily link to major AKI codes and little data is available reflecting their performance against either an adjudicated or histologic standard. Lastly, most administrative coding datasets do not provide a standardized approach to staging of severity outside of recording dialysis. Disease or procedural registries also report data on renal failure, occasionally collecting serum creatinine data. However, definitions for AKI in such registries tend to focus on the most severe phenotypes, may not distinguish between acute or chronic disease, or harmonize with modern consensus definitions. For example, the National Surgical Quality Improvement Program (NSQIP) and American College of Surgeons Committee on Trauma have traditionally defined AKI as a rise in serum creatinine above 2 mg/dl (177 μmol/l) or dialysis and a serum creatinine above 3.5 mg/dl (309 μmol/l), respectively [5–7]. This heterogeneity may hinder comparisons and underestimate disease burden. Recent efforts to recalibrate renal failure definitions, however, may indicate greater acceptance of standardized definitions in some of these data sources. For example, the Society of Thoracic Surgeon database recently changed from a threshold creatinine of > 2 mg/dl (177 μmol/l) to define AKI to incorporate the RIFLE classification system (http://www.sts.org/sites/default/files/documents/Training%20Manual%20for%20website_2.pdf).

**Table 1:** Examples of Administrative codes for AKI

ICD-9-CM	ICD-10	Name
**584**	**N17**	**Acute kidney failure**
**584.5**	**N17.0**	**Acute kidney failure with lesion of tubular necrosis**
**584.6**	**N17.1**	**Acute kidney failure with lesion of renal cortical necrosis**
**584.7**	**N17.2**	**Acute kidney failure of renal medullary (papillary) necrosis**
**584.8**	**N17.8**	**Acute kidney failure with other specified pathological lesion in the kidney**
**584.9**	**N17.9**	**Acute kidney failure, unspecified**
593.9	N28	Unspecified disorder of kidney and ureter
	N28.0	Ischemia and infarction of the kidney
	N28.9	Disorder of kidney and ureter
583.00-90	N00.x N01.x N05.2,5,8,9	ICD- 9: Nephritis and nephropathy, not specified as acute or chronic with lesion of …. ICD-10: Acute Nephritic syndrome (N00) with… Rapidly progressive nephritic syndrome (N01) with Unspecified nephritic syndrome (N05) with…
669.30,32,34	O09.4	ICD-9 Acute kidney failure following labor and delivery…
	N99.8	Postprocedural (acute)(chronic) kidney failure
	O04.82	Renal failure following (induced) termination of pregnancy
	O07.32	Renal Failure following failed attempted termination of pregnancy
	N10	Acute tubulo-interstitial nephritis
	N14.0-4	Drug- and heavy-metal-induced tubule-interstitial and tubular conditions (acuity not specified)

The most commonly studied and used codes are bolded

Coding performance has been examined in several studies by comparing them to a reference standard of creatinine change or manual chart review [8–11]. Vlasschaert et al. performed a systematic review examining the diagnostic accuracy of ICD-9 and ICD-10 codes in US and Canadian datasets between 1987 and 2004 [8]. Despite variations in the reference standards used (varying creatinine-based definitions of AKI or chart review), sensitivity was generally low (median 29 %; range 15-81 %) though specificity remained high (>94 % in all datasets examined). This performance translated to high negative predictive values with variable positive predictive values depending on the cohort examined and underlying prevalence of AKI (between 0.5-52 %). Despite having a lower prevalence of chronic kidney disease, sensitivity in children for AKI is also poor. A recent study found that administrative data had poor sensitivity (21-23 %) for detecting nephrotoxin-associated AKI in children who had serum creatinine monitored daily [12]. Performance between US and Canadian datasets were similar, although sensitivity was slightly higher in Canadian datasets. Restriction to dialysis-requiring AKI generally improved validity, but not always. For example, Waikar et al. found that the combination of any Acute Kidney Failure code (574.5-9) and hemodialysis procedure codes produced high sensitivity (90.4 %) and specificity (93.8 %) for detecting dialysis-requiring AKI as verified by chart review in Boston-area teaching hospitals (USA) between 1988–2002 [13]. However, more recently, Grams et al. used a similar approach to examine the performance of administrative codes for dialysis-requiring AKI in a select cohort of participants of The Atherosclerosis Risk in Communities (ARIC) study hospitalized in Washington County, Maryland between 1996–2008 [11]. In this study, specificity for the dialysis-requiring AKI coding algorithm was high (99.9 %) but had lower sensitivity (36.5 %). Lastly, there is evidence that performance can change over time. For example, several studies have demonstrated that the sensitivity of billing codes has improved in recent years but generally remains modest. [10, 11, 13]

The collective performance of administrative codes for detecting AKI, particularly when not directly linked to serum creatinine, has important implications. For example, poor or changing sensitivities limit value in understanding true disease burden; provide overly conservative estimates of disease prevalence, skew temporal trends due to changes in the sensitivity of diagnostic codes, and contain uncertainty in distinguishing acute from chronic disease. The relative high specificities may make current administrative data more amenable to examining outcomes of patients identified with AKI. However, positive predictive value may vary depending on the underlying risk of the population studied and the severity detected may also vary across regions or time.

*Question #2: What are the limitations, gaps in knowledge, and major barriers to scientific progress in AKI related to how AKI is coded?*

*Consensus Statement 2a: Major limitations of current administrative data include poor awareness of AKI and the subjective nature of its assessment, lack of information on severity, and the use of non-consensus criteria for defining and staging AKI.*

*Consensus Statement 2b: Major knowledge gaps in AKI using current administrative data sources include, but are not limited to, lack of contextual phenotyping, information on complications including dialysis-dependence or other indicators of recovery, and coding structure and performance in non-North American registries.*

### Limitations of administrative data

**Fig. 1:**
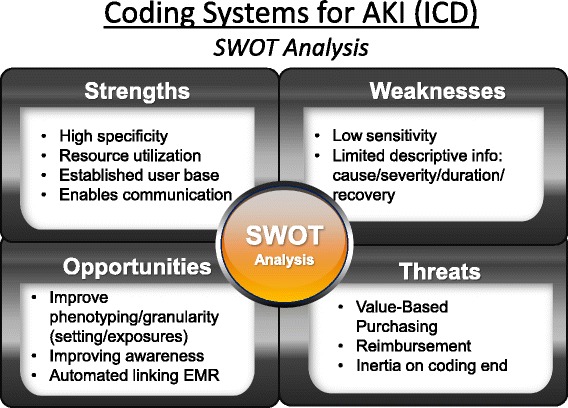
Strength, Weaknesses, Opportunities, and Threat (SWOT) Analysis of Current Administrative Codes for AKI. Source: Acute Dialysis Quality Initiative 15 www.adqi.org; used with permission

Figure 1 shows the current strengths, limitations, opportunities, and threats to optimal use of administrative datasets for examining AKI. In addition to strengths listed, we identified several factors contributing to the limited performance of current administrative coding.

**Fig. 2:**
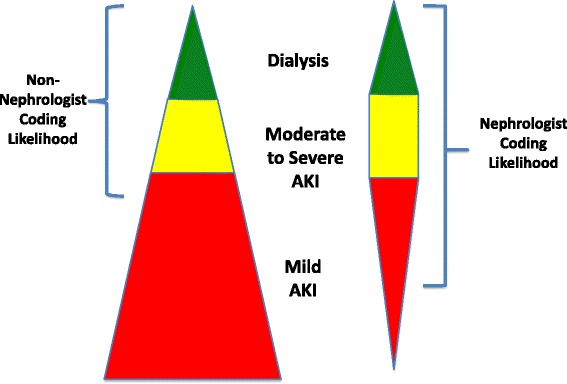
Relative Differences in Scope of AKI Cared and Coded for by Provider Type. The pyramid on the left represents the full burden of AKI seen by non-nephrologists with the relative prevalences of mild, moderate-severe, and dialysis-requiring AKI indicated by shading. In contrast, while nephrologists are more likely to code for less severe AKI, they will encounter a more select population of AKI patients favored by highly severe stages. Source: Acute Dialysis Quality Initiative 15 www.adqi.org; used with permission

One major limitation likely contributing to the low sensitivities observed is poor provider awareness. Accurate and sensitive reporting of AKI depends on recognition that 1) AKI has occurred and 2) it was clinically relevant. As most AKI does not require dialysis and not linked to a procedure code, coding becomes wholly dependent on provider recognition. AKI is generally well-recognized by nephrologists or other experts, however, primary diagnosis codes for patients requiring acute care are most often entered by providers or coding personnel with less expertise or interest in the area. While consensus criteria have likely improved awareness, overall sensitivity remains modest. [11] The resulting discrepancy between exposure and awareness contributes to underreporting of disease (Fig. 2), underscoring the need for improved dissemination of data regarding the short- and long-term clinical implications of AKI to a broader audience [14].

Subjective coding may also be influenced by external forces, including patient related factors (i.e. patient complexity), variations in practice and coding patterns across medical disciplines, geography, and practice settings. For example, capturing AKI in a critically ill patient with prolonged hospitalization, multi-system organ failure, and contributing comorbidities requires overcoming substantial coding ‘inertia’. In addition, while sensitivity has improved, the resulting changes make interpreting temporal trends challenging. For example, uncertainty exists as to whether increases in disease incidence and improvement in survival observed are attributable to better reporting of less severe AKI, changes in case-mix (e.g. more acute on chronic disease), or true improvements in care [13, 15]. Similarly, variations in treatment may affect underlying assumptions in classifying AKI. For example, dialysis-requiring AKI has traditionally been considered the most severe form of AKI. However, thresholds to initiate dialysis may vary depending on situation or over time that can violate that assumption. For example, a mildly volume depleted patient dialyzed for lithium intoxication may not have severe AKI and evidence for earlier application of RRT in recent years may indicate a trend toward dialyzing less severe injury [16]. A lack of readily accompanying information (e.g., indication or stage) in these situations often hinders the ability to make these important distinctions.

### How improving upon coding limitations may help address current knowledge gaps in AKI

It remains possible to enhance administrative data to address critical knowledge gaps in AKI. For example, an increased understanding of the changing case-mix of AKI and its relative impact in different settings is needed [17]. While novel biomarkers may eventually redefine phenotyping, opportunities exist to provide additional contextual information. For example, as AKI most often occurs due to underlying illness, it is most often phenotyped based on the setting in which it occurs. Thus, linking the diagnosis of AKI as a complication of conditions including sepsis (e.g. ‘sepsis complicated by AKI’, ‘contrast/chemotherapy-associated AKI’), might help overcome the inertia of coding AKI as a separate entity and harmonize with how most AKI is conventionally phenotyped (i.e. by clinical context rather than histology).

Further phenotyping of AKI severity and pre-/post- hospitalization kidney function may better understanding of the role of AKI on the natural history of kidney health and disease progression. However, lack of quality information on pre-morbid kidney function, inpatient trajectory, and post-AKI kidney function in current administrative datasets introduce uncertainty into analyses. Integration or linkage of data from advancing electronic health records would enable use of unified diagnostic criteria, distinguishing relative contributions from AKI and CKD, improve further critical phenotyping of AKI (e.g. community-versus-nosocomial acquired AKI, creatinine trajectory, duration, recovery trends), and examine future outcomes.

### Barriers to improving administrative coding

Integrating unified diagnostic criteria and providing contextual information will require some reconfiguration of existing infrastructure. Key stakeholders including payers, government, providers, and coders will need to be engaged and convinced that doing so will improve costs and quality by providing a fuller understanding of disease burden, help to identify patients at high risk for the sequelae of AKI, and opportunities to examine care.

Meaningful changes in coding infrastructure in the kidney disease space are not without precedent. In 2005, the Kidney Disease Outcomes Quality Initiative (KDOQI) staging system for CKD was incorporated into the ICD-10 coding structure. The impetus for these changes in the US was provided by the National Center for Health Statistics, National Kidney Foundation, and the Renal Physicians Association whose goals were to improve understanding of disease burden, follow patients longitudinally, understand treatments provided, and assess the quality of care being delivered. More recent examples of mutual engagement include ongoing initiatives between the critical care community in Canada and the Canadian Institute for Health Information (CIHI) to develop a standardized structure for reporting critical care capacity, operations, quality, and outcomes data. This collaboration will enable a national description of critical care utilization, quality indicators and outcomes aimed at driving health systems improvements. Another current example that model such improvements include the National Health Services ‘Think Kidneys’ program in the United Kingdom [18]. By coupling electronic alerting embedded in all hospitals to improve the recognition of AKI and reduce variability in ascertainment and reporting, developing this critical infrastructure has enabled the building of a prospective registry to capture data on all patients with AKI in a standardized fashion and link to a larger National Renal Registry.

A non-diagnosis related barrier is the deliberate transition of medical records to digital filing. Although the American Reinvestment and Recovery Act of 2009 mandated use of EHRs, integration across institutions in the United States has taken longer than anticipated leading to variability in the extractability of information (as some data remains in paper charting). Additionally, the coding structure and performance for capturing AKI in systems outside of the US, Canada, and UK are underreported. These inconsistencies negatively impact the reliability of administrative datasets across registries and limit the ability to do cross-institutional/-country comparisons.

Finally, a down-stream concern related to changes in coding is the potential for negative reimbursement for ‘nosocomially-acquired AKI’. As value based purchasing and quality-based reimbursements by both insurance agencies and governmental emerge (e.g., US), granularity in AKI diagnosis with regards to the etiology of injury could actually dissuade clinicians from coding for AKI (e.g., contrast nephropathy induced AKI may be deemed a nosocomial AKI and therefore not reimbursed). Avoidance of this problem will require involvement of relevant stakeholders to ensure appropriate quality metrics that do not penalize accurate diagnostic coding.

*Questions #3: What would be the ideal minimal definition for AKI in administrative databases to enable “communication” and “translation” within and across administrative jurisdictions (i.e., institutional, regional, national, international)?*

*Consensus Statement #3: The ideal definition of AKI in administrative databases that would enable cross-registry communication would include descriptors of the onset, duration, severity, context, histology, progression, and recovery of AKI. Minimum essential elements include a standardized, objective description of AKI severity (i.e., stage) accompanied by complementary information on the context in which AKI occurs.*

Definitions of AKI have evolved over the past 20 years as stakeholders have begun to appreciate the benefits of consensus, both for clinical management and for research. The achievement of standardized definitions (e.g., RIFLE, AKIN, KDIGO) has advanced the field by providing a platform for communication and comparison [19–21]. While emerging evidence indicates that clinicians are beginning to use these consensus criteria in quality improvement initiatives [22, 23], widespread evidence of its penetration into administrative datasets is lacking.

**Fig. 3:**
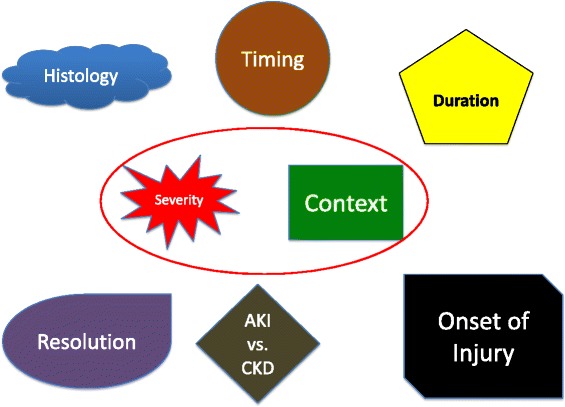
Elements of an “Ideal” Definition of AKI Potentially Captured in Future Administrative Data. An idealized future definition of AKI would include descriptive elements able to identity cause, severity, chronicity, type, timing, and context. Given current limitations in clinical phenotyping and coding structure, severity of injury and the context in which it occurs (e.g. cardiac surgery, sepsis, contrast) may be the best initial targets to pursue. Source: Acute Dialysis Quality Initiative 15 www.adqi.org; used with permission

An ideal “administrative” definition of AKI would incorporate factors that describe the onset, duration, severity, context, histology, progression, and recovery of AKI (Fig. 3). Limitations in current clinical phenotyping and the practicality of standardizing all of these elements as discussed above place some feasibility restrictions on this idealized goal for the near future. However, these limitations do not preclude attainable progress.

Two areas identified where improvements are *currently* possible include improving coding of AKI severity and providing contextual information for the setting in which AKI occurs (Fig. 3 red oval). Introduction of the KDIGO classification system for AKI including “mild” (stage 1), “moderate” (stage 2) and “severe” (stage 3) AKI would enhance capture and phenotyping of AKI beyond the binary system currently in place. As AKI diagnosis relies heavily on laboratory data, the fidelity of these systems will be improved greatly and lend themselves to true standardization through automated inclusion of laboratory data as research networks evolve to integrate large volumes of EHR, administrative, and registry data sources. The latter would also enhance the ability to distinguish between chronicity, onset (community versus hospital-acquired), and permit longitudinal phenotyping including recovery.

Similarly, current AKI coding systems should be modified to provide additional contextual information in which AKI occurs. A natural extension would be to include AKI as a complication of known major precipitants (e.g., sepsis, contrast, cirrhosis, cardiac surgery) as is currently the case for CKD coding algorithms (e.g., diabetes mellitus with diabetic chronic kidney disease, hypertensive chronic kidney disease). As most cases will not be seen by nephrologists, this structure may also have the added benefit of improving sensitivity as clinicians are more likely to consider AKI if linked to the primary disease rather than overcoming the effort to code it as a separate entity.

In summary, limitations in the current clinical phenotyping of AKI should *not* prevent tangible improvements to the current coding of AKI. The reliance of physiologic and laboratory data for AKI diagnosis should prompt progressive consideration for automatic capture and inclusion as administrative datasets evolve.

*Question #4: How can administrative databases be configured to inform “registry-based” pragmatic studies?*

*Consensus Statement #4A: Administrative databases can be used to identify and enrich target populations of interest and inform the feasibility and power of trials.*

*Consensus Statement #4B: Patients enrolled in clinical trials can be potentially linked to administrative databases to enhance outcome ascertainment, reduce costs, and examine additional outcomes of interest if patient identifiers are available.*

**Fig. 4:**
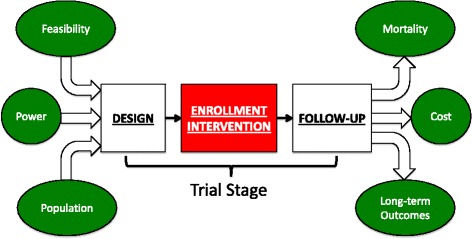
Schematic Illustrating Potential Leveraging of Administrative Data for Clinical Trials. Given limitations in time of reporting and sensitivity, administrative data may be best suited to facilitate the planning and follow-up stages of a putative randomized or pragmatic trial, though disease or procedure-specific registries may be able to be used as a ‘real-time’ tool to guide enrollment or intervention. Source: Acute Dialysis Quality Initiative 15 www.adqi.org; used with permission

Administrative databases, particularly if enhanced by improvements in coding and accuracy, can be leveraged to inform clinical studies or facilitate registry-based pragmatic studies (Fig. 4). First, administrative databases can help inform study design by refining inclusion and exclusion criteria. Analysis of characteristics that identify patients with, or who are likely to develop AKI and its sequelae, can refine the approach to targeting of patients, estimate study feasibility, and project event rates and sample size.

Poor recruitment can hamper trials [24]. Identifying sites with high volumes of patients meeting inclusion criteria allows for targeted collaboration across locations and could improve recruitment. Assessment of event rates can also reduce the need for mid-study adjustments in sample size [25]. The use of large databases for such practices is well illustrated by the design of the PROMISE trial; this trial of early-goal directed therapy for severe sepsis patients in the United Kingdom accurately predicted in-hospital mortality in the control arm based on preliminary assessment of the Intensive Care National Audit and Research Centre (ICNARC) Case Mix Programme Database, a registry of intensive care patients in the United Kingdom [26]. It is important to note that this use of administrative data to inform studies related to AKI requires a clear knowledge of the quality of the data. Datasets with poor sensitivity but high specificity for AKI may be better suited to examine outcomes in populations with moderate to severe AKI rather than those at risk or with mild AKI. Such limitations do not preclude the use of administrative data for these purposes, but do limit the ways they may be used.

Another potential benefit of administrative data in clinical studies is the ability to enhance follow-up of patients already enrolled in studies [27]. Administrative data that provide longitudinal information or can be readily *linked through available identifiers* to other outcome registries (death, ESRD) can be a powerful and cost-saving tool to augment long-term follow up to determine basic outcome information such as mortality, or dialysis. Administrative data can also provide information on other important outcomes such as long-term healthcare resource use by individuals following an intervention as well as cost data. The latter may be an important tool as larger trials are proposed and follow-up of individuals becomes prohibitively expensive, particularly among studies where patients may be more difficult to track using traditional means such as telephone interviews or surveys.

Notably, some administrative databases may be “locked” in terms of the data available in them (e.g. Medicare) that may preclude use for tracking specific outcomes. For example, some select adverse events used as surrogate or part of a composite outcome may require more detailed information for adjudication (e.g. post-operative myocardial infarction) that available. However, other administrative databases may have some flexibility in terms of adding data fields for the purposes of a clinical study. An example of this might be the addition of the type and duration of dialysis in a registry of ICU patients that normally only requires inclusion of a binary variable for dialysis.

Given the current time lag of data availability in most administrative datasets, some may be less well-suited to guide real-time enrollment or randomization of individual patients during the acute phase of illness. This would be especially true where information on short-term events (e.g. hospital mortality) or a greater degree of granularity to accurately capture process-oriented variables such as select adverse events or complications are required in a timely fashion. However, administrative or registry data may be potentially used in certain situations. For example, in a cluster-randomized study where a process of care such as quality improvement or compliance with care bundles (e.g. use of intravenous volume expansion prior to contrast exposure) is an outcome, the use of administrative data may be feasible. More recently developed disease and procedure registries, such as the APPROACH database, a Canadian registry of patients undergoing cardiac catheterization and cardiac surgery, are being used to capture the majority of process and outcome data for the trial. (ClinicalTrials.gov Identifier: NCT02096406). Including research stakeholders within administrative or disease-registry development or oversight teams may facilitate their configuration to integrate research and quality improvement aims.

## Conclusions

In summary, administrative datasets have facilitated the study of AKI on a population-level and are being leveraged by an expanding user base. Major limitations currently include low sensitivity, the subjective nature of assessment, and limited clinical phenotyping of AKI. These limitations highlight important opportunities to improve the quality and coding of data to better address critical knowledge gaps in AKI and improve care. Refinements in coding and the potential to link to increasingly sophisticated EMR data represent key opportunities to advance current administrative data. While an idealized, comprehensive definition of AKI is not currently tenable, tangible progress can currently be made and can help refine how AKI is captured and described within administrative data. We propose the integration of standardized classification schemes and the improvement of linkage of AKI to the clinical context in which it occurs as feasible, substantial improvements on current coding. Progress will require continued efforts to improve awareness of the impact of AKI on public health, engagement of key stakeholders, and tangible strategies to reconfigure infrastructure and improve the reporting and phenotyping of AKI.
